# Associations between early improvements in atherogenic lipids and reported dietary changes after RYGB: a pilot exploratory analysis in women with type 2 diabetes

**DOI:** 10.1016/j.clinsp.2026.101125

**Published:** 2026-09-15

**Authors:** Gabriela de Oliveira Lemos, Ana Paula Aguiar Prudêncio, Renato H. Nakagawa Santos, João Valentini Neto, Aline Marcadenti, Regina Mara Fisberg, Helena Menezes, Steven B. Heymsfield, Raquel Susana Torrinhas, Dan Linetzky Waitzberg

**Affiliations:** a Laboratory of Nutrition and Metabolic Surgery of the Digestive Tract (LIM-35), Department of Gastroenterology, Faculdade de Medicina da Universidade de São Paulo (FMUSP), São Paulo, SP, Brazil; b Instituto de Pesquisa do HCor (IP-Hcor), Associação Beneficente Síria, São Paulo, SP, Brazil; c Department of Nutrition, Faculdade de Saúde Pública da Universidade de São Paulo (FSP/UP), São Paulo, SP, Brazil; d Department of Nutrition, Harvard T. H. Chan School of Public Health, Boston, MA, USA; e Graduate Program in Health Sciences (Cardiology), Instituto de Cardiologia/Fundação Universitária de Cardiologia do Rio Grande do Sul (IC/FUC), Porto Alegre, RS, Brazil; f Graduate Program in Public Health, Faculdade de Saúde Pública, Universidade de São Paulo (FSP-USP), São Paulo, SP, Brazil; g Pennington Biomedical Research Center, Metamor, Baton Rouge, LA, USA

**Keywords:** Food intake, Cholesterol, Lipid profile, Roux-en-Y gastric bypass

## Abstract

**Introduction:**

Roux-en-Y Gastric Bypass (RYGB) is considered a metabolic surgery due to its early effects on obesity-related dysfunctions, including dyslipidemia. However, some studies suggest that these improvements may be reproduced by dietary changes, primarily calorie restriction. We evaluated whether early post-RYGB changes in dietary intake are associated with improvements in the plasma lipid profile in women with Type 2 Diabetes (T2D).

**Methods:**

We analyzed retrospective paired data from women in the SURMetaGIT cohort at baseline and 3-months after RYGB. Dietary intake was assessed by 7-day food records, summarized as F&V (Fruits/legumes/Vegetables) and HFSS (high-fat/sugar/sodium) food groups, and quantified for energy, macronutrients, and fiber using Nutrabem®. Usual intake was estimated using the Multiple Source Method and adjusted for energy. Plasma lipids included total cholesterol, LDL-c, HDL-c, non-HDL-c, VLDL, and triglycerides. Paired comparisons, correlations, and multivariable linear regression were used to evaluate associations between dietary changes and lipid outcomes.

**Results:**

Post-RYGB, patients exhibited a significant decrease in pro-atherogenic lipids. These changes did not correlate with variations in energy, macronutrient, or food-group intake. Conversely, HDL-c showed an independent positive association with phytosterols (β = 0.34, 95% CI: 0.10–0.59; p = 0.007) and a negative association with trans fatty acid intake (β = -0.44, 95% CI:0.82 to -0.06; p = 0.025).

**Conclusion:**

Early pro-atherogenic lipid improvements in women with T2D after RYGB showed no association with changes in reported energy macronutrient or broad food group intake. Nevertheless, trends in HDL-c were associated with intake of phytosterols and trans fatty acids, suggesting a potential role for dietary counseling to optimize HDL-c after RYGB.

## Highlights

• Early after RYGB, atherogenic lipid markers decreased in women with T2DM.
• Early changes in dietary intake did not influence pro-atherogenic lipid particles.
• HDL-c levels were shown to be influenced by phytosterol and trans-fat intake.
• Diet quality should be explored as a lever to optimize HDL-c levels after RYGB.

## Introduction

Diet strongly influences dyslipidemia, affecting cholesterol levels and composition.^1^^,^^2^ As a result, dietary counseling is a key element in guidelines for preventing cardiovascular disease.^3^

Cardiometabolic improvements after Metabolic and Bariatric Surgery (MBS), including Roux-en-Y Gastric Bypass (RYGB), are accompanied by marked reductions in total energy intake and shifts in food selection and diet composition.^4^ In this sense, calorie-matched studies indicate that a substantial proportion of early benefits after MBS ‒ particularly improvements in hepatic insulin sensitivity and glycemic control ‒ can be reproduced by very-low-calorie dietary restriction, suggesting that acute energy deficit may be a major driver of early postoperative changes.^5^^,^^6^

The contribution of postoperative dietary changes to the remodeling of plasma lipid biomarkers after MBS remains incompletely characterized. This gap is particularly relevant because postoperative lipid responses vary substantially and remain incompletely explained.^7^^,^^8^ In the SURMetaGIT cohort, we similarly observed marked variability in improvements in the plasma cholesterol profile, with a subset of patients failing to exhibit meaningful lipid improvement after surgery.^9^ The extent to which early postoperative dietary changes account for this inter-individual variability ‒ and which dietary dimensions best capture it ‒ remains unclear.

This study tested associations between early lipid remodeling and early changes in dietary intake after RYGB in women with type 2 diabetes from the SURMetaGIT cohort. Primary outcomes were changes in LDL-c and triglycerides because they represent clinically actionable atherogenic targets and are the lipid fractions most plausibly linked to diet composition ‒ particularly fat quality and carbohydrate-related hepatic VLDL dynamics. Secondary outcomes included total cholesterol, HDL-c, VLDL-c, and non-HDL-c.

## Materials and methods

### Study design and population

The present study enrolled 30-adult women (aged 18‒60 years) who underwent RYGB between April 2012 and March 2016 at the Bariatric and Metabolic Surgery Unit of the Hospital das Clínicas, Faculty of Medicine, University of São Paulo (HC-FMUSP). Inclusion criteria included female candidates for MBS diagnosed with T2DM and a body mass index between 35 and 50 kg/m^2^. Exclusion criteria included: use of insulin, severe hepatic dysfunction, thyroid dysfunction, presence of an abdominal or umbilical hernia, *H. pylori* gastric infection, and enrollment in other interventional research protocols. In addition, patients with missing data on either dietary intake or lipid profile before or after surgery were excluded. Only women were enrolled after a specific metabolic and bariatric surgery procedure (RYGB) was performed to reduce data heterogeneity.

The present protocol used a convenience sample from the SURMetaGIT cohort. The original sample size included twenty women, based on transcriptomic outcomes, as described by Sala et al. (2016),^10^ with 10 additional participants enrolled for gene expression, bringing the total to 30 patients. The sample size estimation for the SURMetaGIT study is fully described elsewhere.^10^ Patients included in the study underwent dietary intake analyses and plasma/serum sample collection procedures at the time of study inclusion and 3-months after the surgical procedure.

### Plasma and serum samples

Plasma and serum samples were obtained from peripheral blood for biochemical analysis. Total cholesterol and its fractions were measured using the colorimetric enzymatic CHOD/PAP (Cholesterol Oxidase/Peroxidase-Aminoantipyrine) method. All blood collections were performed after a 12-hour fast. The collected samples were centrifuged at 2800 rpm and 4 °C, aliquoted into 500 μL microtubes, and stored at −80 °C until biochemical analysis.

### Roux-en-Y gastric bypass

All patients underwent the RYGB surgical technique at the Bariatric and Metabolic Surgery Unit (HC-FMUSP) via laparotomy. In the present study, the gastric pouch had a capacity of approximately 30 mL, and the lengths of the biliopancreatic and alimentary limbs were standardized at 50‒60 cm and 100‒120 cm, respectively.

### Study’s variables

#### Participant characterization

We assessed demographic and Lipid-Lowering Therapy (LLT) data. Type 2 Diabetes (T2DM) was defined according to the American Diabetes Association criteria^11^ as FPG ≥126 mg/dL and HbA1c ≥6.5% or use of oral antidiabetic medication. Recorded data on the plasma lipid profile, including Total Cholesterol (TC), High-Density Lipoprotein (HDL) cholesterol, Low-Density Lipoprotein (LDL) cholesterol, Very-Low-Density Lipoprotein (VLDL) cholesterol, triglycerides, and non-HDL cholesterol, were collected. Body weight (kg) and height (cm) were measured using an electronic scale (Lucastec®, Brazil) and a stadiometer (Sanny®, American Medical of Brazil), respectively. Body Mass Index (BMI) was calculated as body weight (in kilograms) divided by height squared (in meters squared).

#### Dietary intake analysis

Dietary intake was assessed using 7-day Food Records (7dFR) collected before and 3-months after the surgical procedure. Reported foods were recorded in household measures (e.g., tablespoons), with the aid of illustrations from a standardized manual provided to all participants.^12^ Completed food records were reviewed by trained nutrition researchers for completeness and internal consistency, and participants were contacted whenever clarification was required. Subsequently, the research team converted these measures into grams or milliliters, using a standardization book for household measures.^13^ Additionally, records with implausible energy intakes (< 500 or > 3500 kcal/day for women) were excluded from the analyses.

Participants were instructed to complete the 7-day food records during the same week as the clinical and laboratory assessments at both the preoperative and 3-month postoperative visits. Thus, dietary intake was assessed as close as possible to the blood collection time points, minimizing temporal variability between dietary and biochemical measurements.

Foods were categorized into two groups based on nutritional recommendations for managing dyslipidemia. The first group was the F&L (fruits, vegetables, and legumes), and included components known as health-protective, such as fruits, non-starchy vegetables, and leafy greens, whereas the second group was the HFSS (high-fat/sugar/sodium), which included components known as non-protective for health, such as foods high in saturated fats, sodium, and added sugars (Supplementary S1), as defined by Brazilian nutritional labeling legislation.^14^^,^^15^

The intake of total energy, macronutrients, fiber, fatty acids, phytosterols, and foods was estimated using the Nutrabem^®ฏ^ software.^16^ Estimation of usual energy and nutrient intake was performed using the Multiple Source Method (MSM), which used a specific online platform for this purpose.^17^ All nutritional variables and food groups were adjusted for total energy intake using the residual method.^18^ In addition, all statistical analyses were performed after proper energy adjustments using the residual method, and the results were presented accordingly.

### Statistical analysis

Changes in dietary intake and plasma lipids are described as ratios of post-operative to pre-operative values. Paired food intake analysis was performed for participants with both pre- and post-operative dietary intake data. We assessed the association between changes in dietary intake and changes in plasma lipids, rather than absolute pre- or postsurgical values, to better capture the impact of behavioral shifts over time. This approach is consistent with established methods for evaluating health-related changes in longitudinal cohorts.^19^ Continuous variables are presented as mean and standard deviation when symmetrical, and median and interquartile range when asymmetrical, while categorical variables are presented as absolute and relative frequencies.

Intra-group comparisons were assessed using a paired *t*-test or a Wilcoxon test, depending on whether the data were normally distributed, as determined by the Shapiro-Wilk test. The correlations between changes in food and nutrient intake and changes in the lipid profile were assessed using Pearson's correlation coefficient. Given the small sample size and the exploratory, hypothesis-generating nature of this study, a significance threshold of p < 0.05 was adopted to reduce the risk of excessive Type II error, which could obscure relevant preliminary associations. Significant correlations were further examined using linear regression models adjusted for age, baseline BMI, and changes in LLT use to assess potential associations. For this model, only participants with plasma lipid analysis were included without data imputation. Tests were performed using R software, version 4.4.1.

### Ethical research procedures

The present study is an exploratory analysis of the SURMetaGIT study, an umbrella project approved by the Human Research Ethics Committee of the Faculty of Medicine of the University of São Paulo (HU FMUSP), registered on Plataforma Brazil under number 19339913.0.0000.0068 and on ClinicalTrials.gov under number NCT01251016. It was approved by the Local Ethics Committee and conducted in accordance with the Declaration of Helsinki and the Resolution 466/2012 of the National Health Council/Ministry of Health (CNS/MS). In addition, the protocol adhered to the STROBE Statement for cohort studies. We provided information about the research objectives and methodology during the formal invitation period for study participation and/or whenever necessary. Additionally, participants were free to withdraw from the study at any time. Thus, only patients who agreed and signed the Free and Informed Consent Form participated in the study.

## Results

### Descriptive data

The flow diagram of participant inclusion is shown in Appendix A. Dietary intake (n = 29) and lipid profile (n = 28) were assessed in the SURMetaGIT study at baseline and three months after surgery. One participant had incomplete lipid data at follow-up and was excluded from lipid analyses but included in dietary analyses. The baseline characteristics of patients are presented in Table 1.

**Table 1:** Baseline characteristics.

**Variable**	**Value**
Age (years)	47.3 ± 6.9
Weight (kg)	113.9 ± 15.2
BMI (kg/m^2^)	45.6 ± 5.7
Total Cholesterol (mg/dL)	192.5 (175.2; 205.2)
HDL-c (mg/dL)	44.4 ± 9.8
LDL-c (mg/dL)	118.4 ± 34.7
VLDL-c (mg/dL)	29.2 ± 9.3
Triglycerides (mg/dL)	146.0 (108.8; 189.2)
Non-HDL-c (mg/dL)	143.0 (132.8; 165.0)
Energy (Kcal)	1609.7 ± 346.9
F&V (g)	306.4 ± 108.6
HFSS (g)	275.9 ± 101.2
Total lipids (g)^a^	49.9 (46.6; 57.3)
Protein (g)	73.4 ± 7.4
Carbohydrate (g)	199.4 ± 17.7
Fiber (g)	15.7 ± 3.9
Monounsaturated fatty acid (g)^a^	19.7 (17.6; 23.3)
Saturated fatty acid (g)^a^	19.8 (18.0; 21.6)
Trans fatty acid (g)^a^	0.7 (0.6; 0.8)
Cholesterol (g)	247.5 ± 41.2
Polyunsaturated fatty acid (g)^a^	10.0 (8.5; 11.5)
Phytoesterols (mg)	28.0 ± 11.0

HDL-c, High-Density Lipoprotein; LDL-c, Low-Density Lipoprotein; VLDL, Very-Low-Density Lipoprotein.

a Data are shown as the mean ± standard deviation, or median (interquartile range), according to the normality of data.

Patients had an average age of 47-years and a mean weight loss of 18.4%. Before surgery, the average energy intake was 14.1 Kcal/kg per actual body weight. The amount of carbohydrates, lipids, and proteins represented 50.1%, 31.4%, and 18.5%, respectively. Saturated and trans fatty acids accounted for 12.4% and 4.4% of the total energy intake, respectively.

### Plasma lipid changes

After RYGB, total cholesterol levels decreased by an average of 22.3%, primarily at the expense of non-HDL cholesterol: 143.0 mg/dL (132.8‒165.0 mg/dL) vs. 109.5 mg/dL (91.0‒131.0 mg/dL), p = 0.002, but only 10 patients reached levels < 100 mg/dL (35.7%), and 8 remaining ≥ 130 mg/dL (28.6%). HDL cholesterol levels remained low without significant changes (44.4 ± 9.8 mg/dL vs. 42.1 ± 9.5 mg/dL, p = 0.185) (Table 2), and the levels of HDL-c ≥ 50 mg/dL were present in only 7 individuals pre- and post-surgery.

**Table 2:** Lipid biomarkers before and after RYGB.

**Variable**	**Pre (28)**	**Post (28)**	**p-value**^a^
Total Cholesterol mg/dL	192.5 (175.2; 205.2)	149.5 (137.0; 166.2)	**0.001**
HDL-c mg/dL	44.4 ± 9.8	42.1 ± 9.5	0.185
LDL-c mg/dL	118.4 ± 34.7	97.2 ± 37.9	**0.010**
VLDL-c mg/dL	29.2 ± 9.3	22.5 ± 9.6	**0.002**
Triglycerides mg/dL	146.0 (108.8; 189.2)	104.5 (91.8; 128.5)	**0.001**
Non-HDL-c	143.0 (132.8; 165.0)	109.5 (91.0; 131.0)	**0.002**

HDL-c, High-Density Lipoprotein; LDL-c, Low-Density Lipoprotein; VLDL, Very-Low-Density Lipoprotein. Data are shown as the mean ± standard deviation, or median (interquartile range).

a Paired *t*-test or Wilcoxon test. Bold numbers represent results with statistical significance (p < 0.05).

The use of LLT before and after surgery is described in Table 3. Eighteen patients (60%) did not take LLT before or after surgery. Nine patients were weaned off their medication (30%); one patient had their dose decreased (from 120 mg to 20 mg); and three patients (10%) were kept on the same regimen. Overall, 10 (33%) patients underwent changes in their LLT.

**Table 3:** Lipid-lowering therapy before and after surgery.

**Pre-surgery**	**Post-surgery**		**Total**	**p-value**^a^
	**No**	**Yes**		
No	18 (60%)	0 (0%)	18 (60%)	
Yes	9 (30%)	3 (10%)	12 (40%)	
Total	27 (90%)	3 (10%)	30 (100%)	0.008

a p-value was calculated using McNemar’s test.

At baseline, 6 patients (21.4%) had LDL cholesterol levels ≤100 mg/dL, and 2 patients (7.1%) had levels ≤ 70 mg/dL. After surgery, these numbers increased to 20 (71%) and 6 (21%), respectively. Four patients exhibited high LDL cholesterol levels (> 100 mg/dL) due to a post-procedure increase; all of them had discontinued LLT. Regarding triglyceride levels, 13 (46%) and 4 (14%) patients had plasma levels ≥ 150 mg/dL pre- and post-operatively, respectively. Two patients developed high triglyceride levels (> 150 mg/dL) following the procedure, and both had also discontinued LLT, which was reported in 12 patients at baseline and in 3 patients after surgery.

The changes in lipid markers did not correlate with changes in body weight or BMI, except for triglycerides, which showed a moderate positive correlation (*r* = 0.43, p = 0.02).

### Food and nutrient intake changes

**Fig. 1:**
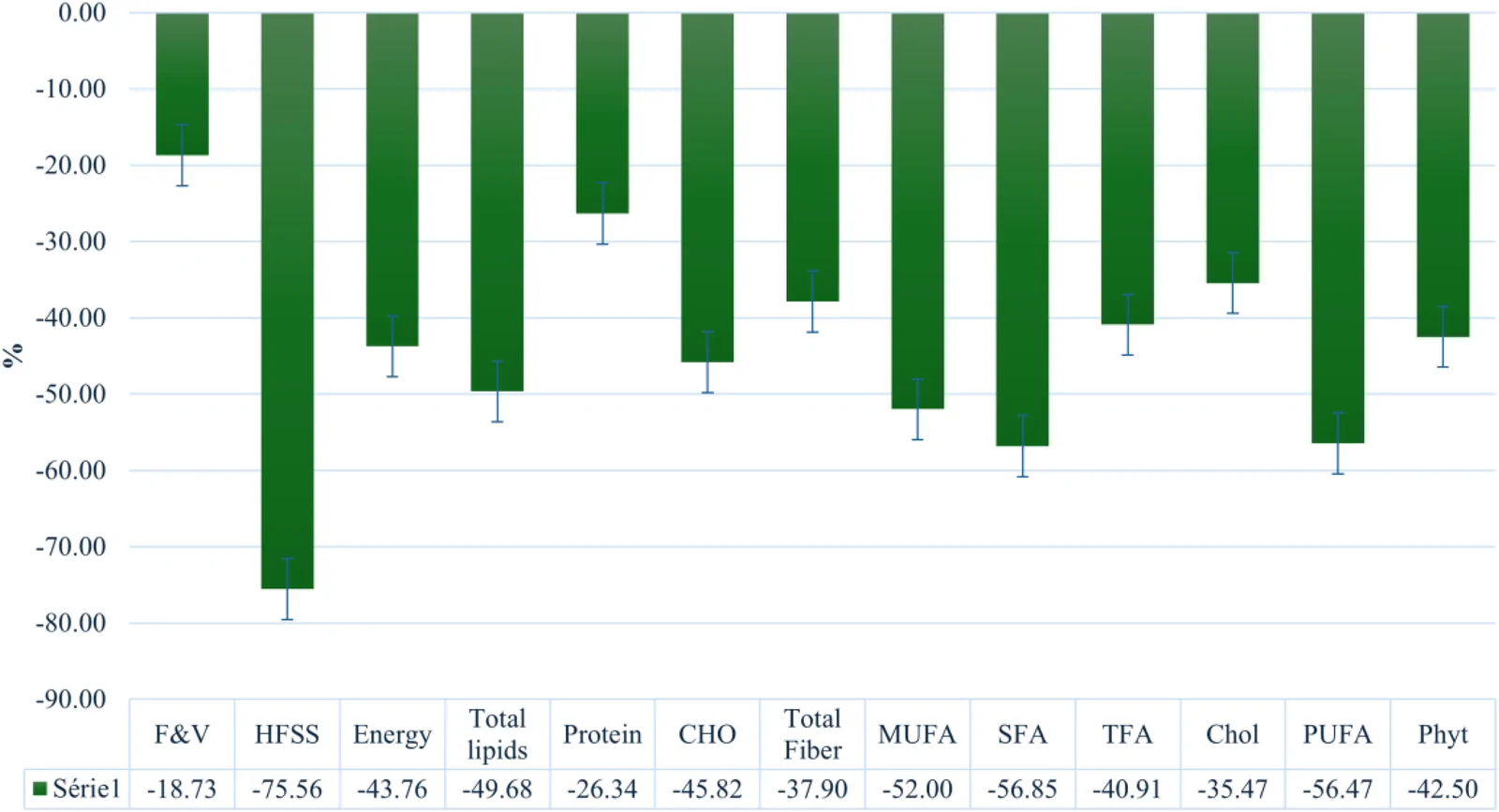
Dietary intake changes after RYGB. CHO, Carbohydrates; Chol, Cholesterol; F&V, Fruits and Vegetables; HFSS, Foods High in Fat, Sugar, and Salt; MUFA, Monounsaturated Fatty Acids; Phyt, Phytosterols; PUFA, Polyunsaturated Fatty Acids; TFA, Trans Fatty Acids.

Energy intake decreased by 43.8% (from 1609.7 ± 346.9 Kcal to 893.6 ± 213.4 Kcal, p < 0.001). Changes in energy intake after surgery showed only a trend toward correlation with changes in triglycerides (*r* = 0.36, p = 0.06), which may reflect insufficient statistical power; specifically, the observed power to detect a moderate correlation (*r* = 0.40) with the current sample size (n = 28) is approximately 57,6%. In parallel, the intake of both food groups and all analyzed nutrients showed a significant decrease, particularly the consumption of lipids (49.9 g [46.6‒57.3 g] vs. 26.6 g [23.3‒28.9 g], p < 0.001), with Saturated Fatty Acids ‒ SFA (19.8 g [18.0‒21.6 g] vs. 9.1 g [8.2‒10.5 g], p < 0.001) and Polyunsaturated Fatty Acids ‒ PUFA (10.0 g [8.5‒11.5 g] vs. 4.8 g [4.2‒5.3 g], p < 0.001) representing the most significant changes, and trans fatty acids the least (0.7 g [0.6‒0.8 g] vs. 0.4 g [0.3‒0.5 g], p < 0.001). Among the macronutrients analyzed, the changes in protein intake were the least pronounced (73.4 ± 7.4 g vs. 53.8 ± 8.4 g, p < 0.001) (Fig. 1).

Although absolute intake of F&V showed a statistically significant reduction in the postoperative period [mean (SD): 306.3 (109.7) g vs. 247.8 (92.8) g; p = 0.021], this decrease was proportionally smaller than that observed for total energy intake.

When F&V density was expressed relative to total dietary energy, an approximately 46% increase was observed, rising from 190 g/1000 kcal preoperatively to 277 *g*/1000 kcal postoperatively. In contrast, HFSS intake declined markedly both in absolute terms [median (IQR): 252.0 g (180.5–419.4 g) vs. 65.6 g (44.9–81.9 g); p < 0.001] and proportionally, indicating a substantial reduction in the energy density of these foods within the postoperative dietary pattern.

### Association between food groups and nutrients with the plasma lipid biomarkers

**Fig. 2:**
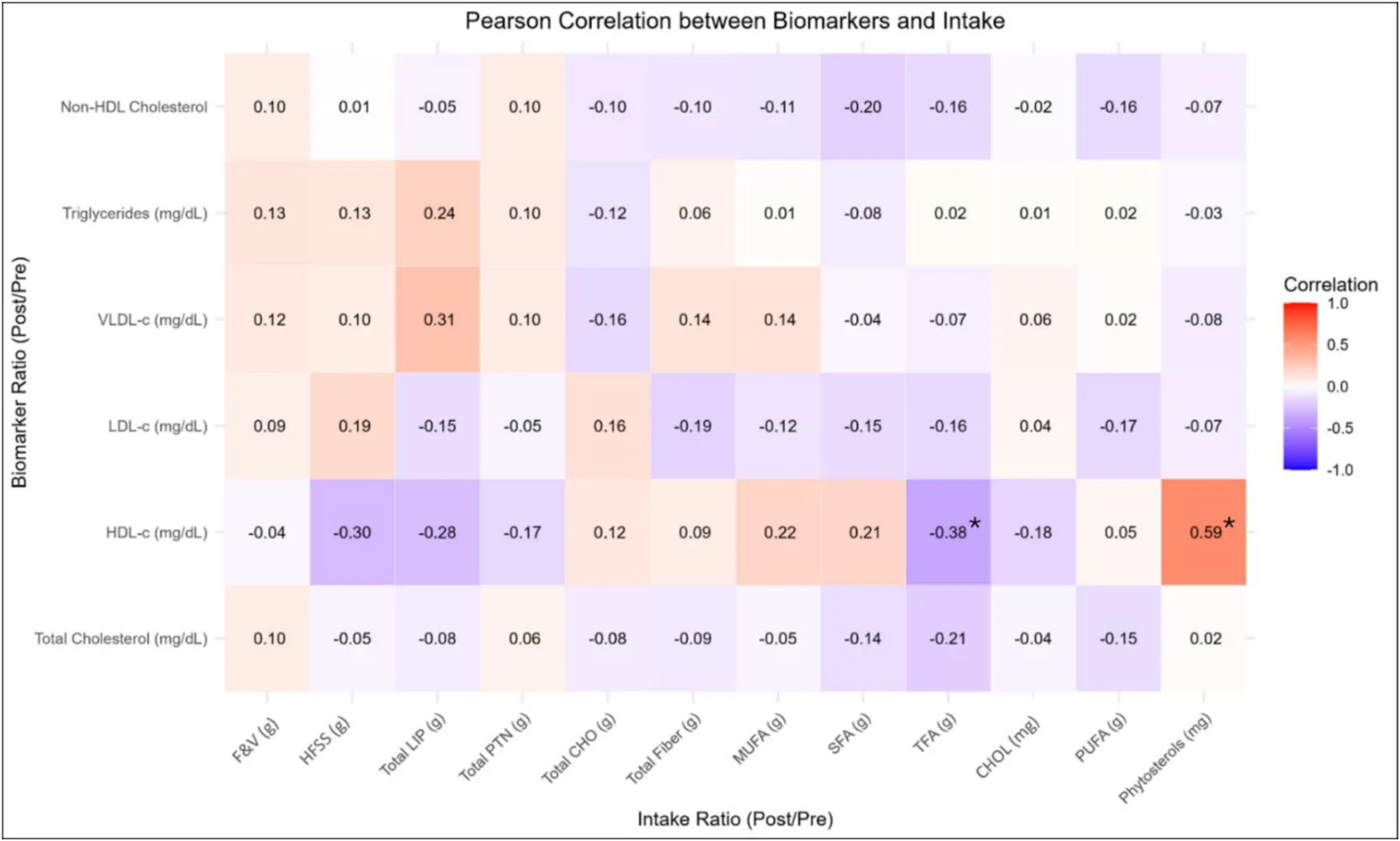
Association between diet and the plasma lipid profile. CHO, Carbohydrates; CHOL, Cholesterol; F&V, Fruits and Vegetables; HFSS, Foods High in Fat, Sugar, and Salt; MUFA, Monounsaturated Fatty Acids; PUFA, Polyunsaturated Fatty Acids; TFA, Trans Fatty Acids; HDL-c, High-Density Lipoprotein; LDL-c, Low-Density Lipoprotein; VLDL, Very-Low-Density Lipoprotein. Values are Pearson’s correlation coefficients. Negative correlations are shown in red, and positive correlations in blue. The more intense the colors in the boxes, the stronger the correlations (* p < 0.05).

The association between plasma lipids and the food groups, as well as the nutrients analyzed, is expressed in Fig. 2. For the primary outcomes, changes in LDL-c and triglycerides did not correlate significantly with any dietary intake variable (all p > 0.05) (Appendix B).

**Fig. 3:**
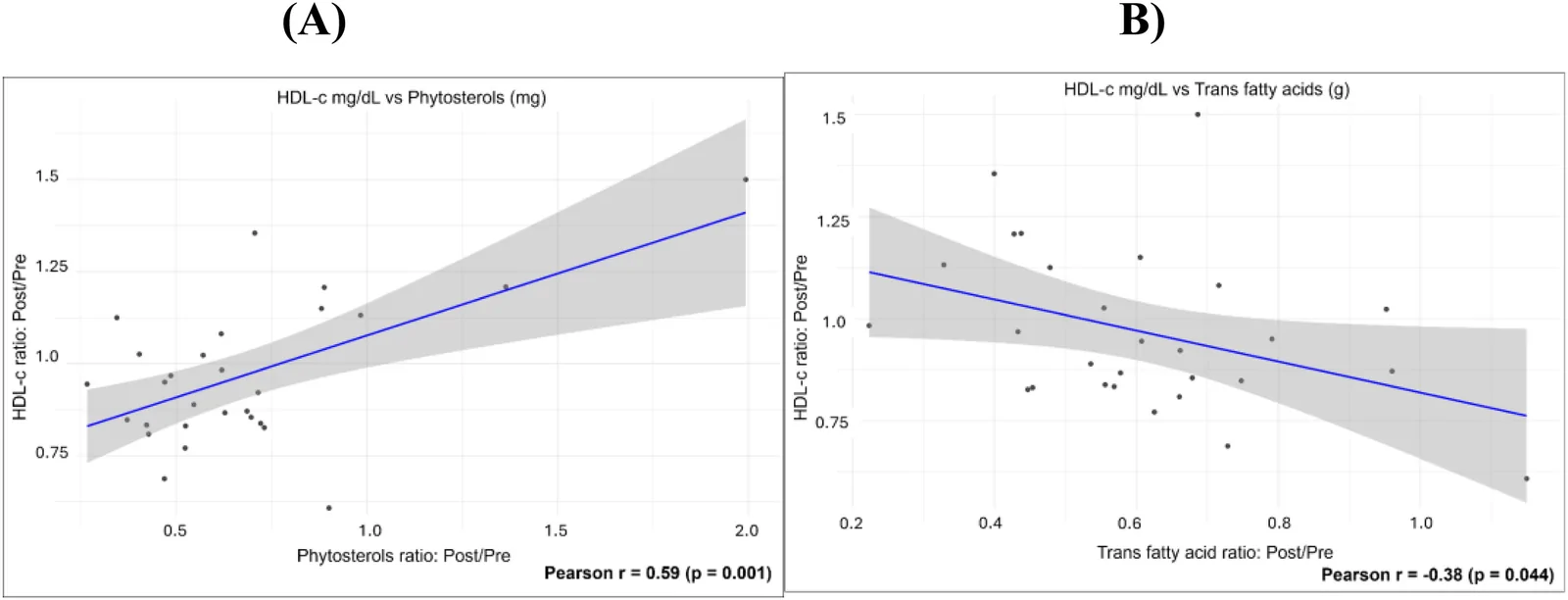
Correlation analysis of phytosterols and trans fatty acid intake with HDL cholesterol. (A) Pearson’s correlation analysis showing a positive association between phytosterol intake and HDL-C levels; (B) Pearson’s correlation analysis showing a negative association between trans fatty acid intake and HDL-C levels. HDL-c, High-Density Lipoprotein.

Regarding the remaining plasma lipid fractions, only HDL-c showed a positive correlation with phytosterol intake (*r* = 0.59, p < 0.01) and a negative correlation with trans fatty acid intake (*r* = −0.38, p = 0.04) (Fig. 3A and 3B).

These associations were examined using linear regression models adjusted for age, baseline BMI, and LLT use. Higher phytosterol intake was positively associated with HDL-c (β = 0.33, 95% CI: 0.10‒0.56; p = 0.007), independent of covariates. In contrast, trans fatty acid intake was inversely associated with the HDL-c ratio (β = −0.42, 95% CI: −0.80 to −0.04; p = 0.031). In both models, age, baseline BMI, and change in the LLT were not significantly associated with the outcome (Appendix C).

## Discussion

Participants in the SURMetaGIT cohort had obesity and T2DM, placing them at elevated cardiovascular risk and underscoring the relevance of stringent lipid targets in primary prevention (e.g., LDL-c < 70 mg/dL or non-HDL-c < 100 mg/dL).^3^^,^^19^

Nevertheless, even with early postoperative improvements, not all participants achieved these targets, reinforcing the need to identify modifiable drivers of residual risk.

MBS reduces cardiovascular risk and improves plasma lipid profiles, yet the drivers of early postoperative lipid remodeling remain incompletely understood.^7^^,^^8^ In our analysis, we observed early reductions in atherogenic lipid measures 3-months after RYGB, whereas HDL-c did not change at the group level.

In parallel, reported energy and nutrient intake decreased markedly, and broad food-group patterns suggested an overall improvement in diet quality, with an increased relative contribution of fruits, legumes, and vegetables and a marked reduction in the contribution of ultra-processed foods. However, these dietary changes were not clearly associated with the magnitude of improvement in atherogenic lipids. By contrast, changes in HDL-c were positively associated with phytosterol intake and inversely associated with trans-fat intake after adjustment for covariates. Although biologically plausible, these findings should be interpreted as independent associations rather than evidence of causality.

Early postoperative shifts in dietary intake would be expected to track changes in atherogenic lipid measures, since dietary intake is markedly reduced after surgery^21^ and is a major determinant of dyslipidemia.^1^^,^^2^ In this small cohort, such associations were not clearly detected among the assessed food groups and nutrients, and changes in energy intake showed only a trend toward association with triglycerides. These findings suggest that early lipid improvements after RYGB may be driven by procedure-related mechanisms and/or metabolic adaptations not captured by self-reported dietary intake during the early postoperative period.

An alternative potential explanation for early lipid improvements after RYGB is diet-related malabsorption. In the short term, however, this mechanism may make only a limited contribution to overall changes in energy absorption, as RYGB has been estimated to account for Only ∼6% of the total reduction in energy absorption during early follow-up.^20^ In addition, comparative evidence suggests that improvements in lipid profiles after malabsorptive procedures may be more closely related to changes in cholesterol absorption and synthesis than to reported dietary intake.^21^

A second, increasingly supported, alternative pathway involves surgery-driven effects on bile acid metabolism and related signaling. After RYGB, enhanced enterohepatic bile acid circulation has been reported, including increased total and primary plasma bile acids and decreased fecal bile salts.^22^, ^23^, ^24^ Conjugated bile acids regulate cholesterol metabolism through intestinal FXR and TGR5 signaling,^25^ providing a plausible mechanistic bridge between gastrointestinal anatomical changes and systemic lipid remodeling.

While both energy intake and diet composition may influence bile acid synthesis (e.g., fat intake stimulates synthesis and fiber intake inhibits production),^26^^,^^27^ the overall pattern of early atherogenic lipid improvements in our cohort ‒ without clear associations with broad measures of reported intake ‒ suggests a substantial contribution of procedure-related mechanisms, potentially overlapping with caloric restriction at some points but not fully captured by intake changes measured here.

Consistent with this interpretation, intestinal gene expression data from the same SURMetaGIT cohort showed that pathways involved in cholesterol and bile acid metabolism were among the most significantly modified in the duodenum and jejunum, including the Superpathway of Cholesterol Biosynthesis, LXR/RXR Activation, and multiple cholesterol biosynthesis pathways.^28^ Together, these observations suggest a model in which early atherogenic lipid improvements after RYGB reflect, to a meaningful extent, surgery-driven regulation of cholesterol and bile acid pathways.

Only a limited number of studies have directly compared calorie restriction and MBS with respect to lipid outcomes. Saiyalam et al. (2014) compared a Very-Low-Calorie Diet (VLCD) versus RYGB over 12-weeks and reported no differences in biochemical profiles, although baseline differences between groups (e.g., age and T2DM prevalence) limited comparability.^29^ Herzog et al. (2020) evaluated whether RYGB exerts additional effects on plasma lipid metabolites after a 6-week very-low-calorie diet and did not identify an additional surgical effect on the metabolites analyzed, supporting a prominent role for calorie restriction in early metabolic improvement.^6^ This interpretation does not fully align with our results; however, Herzog et al. did not include an objective assessment of traditional biochemical lipid markers (total cholesterol and fractions), limiting direct comparison and leaving open the question of whether classic atherogenic lipid measures behave similarly to lipid metabolites early after surgery.

In contrast to the overall pattern for atherogenic lipid measures, the observed associations between HDL-c and specific dietary components merit attention. Although HDL-c did not change significantly at the group level, individual variability in HDL-c was positively associated with phytosterol intake and inversely associated with trans-fat intake after covariate adjustment. The positive association between phytosterol intake and the change in HDL-c was unexpected, as phytosterols primarily lower LDL-c. This finding may reflect residual confounding by overall diet quality, rather than a direct causal effect, since phytosterols are typically consumed alongside other beneficial dietary components; however, a chance observation due to multiple comparisons cannot be disregarded. HDL functionality and quality were not assessed in this protocol (e.g., Cholesterol Efflux Capacity [CEC] was not measured), which limits mechanistic inference and should be assessed in future studies. These findings suggest that while broad dietary changes may not drive early improvements in atherogenic lipid profiles, attention to specific dietary components, such as minimizing trans-fat intake, may still be warranted to optimize HDL-c levels in the postoperative period.

Several limitations should be acknowledged. The small sample size, which was not originally powered for this exploratory analysis, may have limited power, particularly to detect associations between changes in dietary intake and pro-atherogenic lipid particles. In addition, although the 7-day food record is a detailed prospective method for dietary assessment, dietary intake was self-reported, and measurement error cannot be excluded, especially in individuals with obesity. Additionally, the accuracy of self-reported dietary intake may differ before and after surgery due to changes in eating patterns and social desirability bias, potentially affecting estimates of change. The short follow-up period also limits interpretation, as the observed findings may reflect transient postoperative adaptations rather than long-term metabolic changes. Although the inclusion of only women from a single center increased cohort homogeneity, it limits the generalizability of the results. Missing data and the applied exclusion criteria may also have introduced selection bias, and the absence of a non-surgical control group precludes definitive attribution of the observed lipid changes to RYGB. Finally, because multiple associations were tested without formal correction for multiple comparisons, the possibility of chance findings cannot be excluded. Therefore, the observed associations should be interpreted as independent, hypothesis-generating findings rather than evidence of causality.

## Conclusion

In conclusion, this exploratory analysis showed significant early improvements in the atherogenic lipid profile of women with type 2 diabetes after RYGB. These changes were not significantly associated with reported changes in energy or macronutrient intake, suggesting that early lipid improvement may involve surgery-specific metabolic mechanisms rather than dietary modifications alone. In contrast, changes in HDL-c showed associations with phytosterols and trans-fat intake that remained after adjustment for covariates, but should be interpreted with caution given the exploratory nature of the analysis and the lack of correction for multiple comparisons. These preliminary findings support the need for larger studies with objective dietary markers and longer follow-up to clarify the complex interplay between diet, surgical intervention, and long-term lipid metabolism.

## Glossary

7dFR, Seven-day Food Record; ADA, American Diabetes Association; BMI, Body Mass Index; CEC, Cholesterol Efflux Capacity; FPG, Fasting Plasma Glucose; F&V, Fruits, legumes, and Vegetables; HbA1c, Glycated hemoglobin; HDL-c, High-Density Lipoprotein; HFSS, Foods rich in saturated Fat, Sugar, and Sodium; LDL-c, Low-Density Lipoprotein; LLT, Lipid-Lowering Therapy; MBS, Metabolic and Bariatric Surgery; Phyt, Phytosterols; RYGB, Roux-en-Y Gastric Bypass; T2DM, Type 2 Diabetes Mellitus; VLDL-c, Very-Low-Density Lipoprotein.

## Institutional review board statement

This protocol study was conducted in accordance with the Declaration of Helsinki and was approved by the local ethics committee (Comissão de Ética para Análise de Projetos de Pesquisa ‒ CAPPesq). Approval number: 6.054.077; Approval date: May 11th, 2023.

## Authors’ contributions

Gabriela de Oliveira Lemos contributed to the study conceptualization and design, data analysis tools, and wrote the original draft; Ana Paula Aguiar Prudêncio contributed to data collection and curation, and critically reviewed the paper; Renato Hideo Nakagawa Santos contributed to statistical analysis and critically reviewed the paper; João Valentini Neto contributed to data curation, methodology, and reviewed the paper; Helena Menezes contributed to data collection and curation, and critically reviewed the paper; Regina Mara Fisberg contributed to data curation, methodology, and critically reviewed the paper; Aline Marcadenti and Steven B. Heymsfield critically reviewed the paper; Raquel Susana Matos Torrinhas: contributed to the study design and analysis, project supervision, and critically reviewed the paper; Dan Linetzky Waitzberg: conceived and designed the analysis, consistent with other authors, funding acquisition, project administration, and supervision, and critically reviewed the paper. All authors approved the final version of the paper.

## Funding

This study is linked to project n° 2011/09612-3 and was funded by the Fundação de Amparo à Pesquisa do Estado de São Paulo (FAPESP).

## Declaration of competing interest

The authors declare no conflicts of interest.

## Data Availability

The data are available upon request.

## Appendix Supplementary materials

Appendix A: Participant flowchart; Appendix B: Correlation analysis between food intake and plasma lipid biomarkers; Appendix C: Linear regression model for HDL-c changes (post/pre ratio) considering adjusted trans fatty acids and phytosterols intake to age, BMI, and lipid-lowering therapy before and after surgery.
