# Supplementary material for: Associations between early improvements in atherogenic lipids and reported dietary changes after RYGB: a pilot exploratory analysis in women with type 2 diabetes
Source: Clinics (Sao Paulo). 2026 Sep 15;81:101125. doi: 10.1016/j.clinsp.2026.101125 (PMC13594641; doi:10.1016/j.clinsp.2026.101125)
Supplement: Supplementary file 1 [file mmc1.docx]

CLINICS-D-26-00366_Supplementary Material

**Appendix A** Flow diagram for study participants. 7dFR, Seven-Day Food Records; RYGB, Roux-en-Y Gastric Bypass.

**Appendix B** Correlation analysis between food intake and plasma lipid biomarkers.

| **Lipid markers** | **Food intake** | **Pearson’s correlation** | **p-value** |
| --- | --- | --- | --- |
| Total Cholesterol mg/dL | F&V (g) | 0.10 | 0.63 |
| HDL-c mg/dL | F&V (g) | -0.04 | 0.84 |
| LDL-c mg/dL | F&V (g) | 0.09 | 0.66 |
| VLDL-c mg/dL | F&V (g) | 0.12 | 0.57 |
| Triglycerides mg/dL | F&V (g) | 0.13 | 0.50 |
| Non-HDL-c mg/dL | F&V (g) | 0.10 | 0.62 |
| Total Cholesterol mg/dL | LUPA (g) | -0.05 | 0.80 |
| HDL-c mg/dL | LUPA (g) | -0.30 | 0.12 |
| LDL-c mg/dL | LUPA (g) | 0.19 | 0.33 |
| VLDL-c mg/dL | LUPA (g) | 0.10 | 0.64 |
| Triglycerides mg/dL | LUPA (g) | 0.13 | 0.52 |
| Non-HDL-c mg/dL | LUPA (g) | 0.01 | 0.96 |
| Total Cholesterol mg/dL | Total lipids (g) | -0.08 | 0.68 |
| HDL-c mg/dL | Total lipids (g) | -0.28 | 0.15 |
| LDL-c mg/dL | Total lipids (g) | -0.15 | 0.46 |
| VLDL-c mg/dL | Total lipids (g) | 0.31 | 0.12 |
| Triglycerides mg/dL | Total lipids (g) | 0.24 | 0.22 |
| Non-HDL-c mg/dL | Total lipids (g) | -0.05 | 0.79 |
| Total Cholesterol mg/dL | Total protein (g) | 0.06 | 0.75 |
| HDL-c mg/dL | Total protein (g) | -0.17 | 0.40 |
| LDL-c mg/dL | Total protein (g) | -0.05 | 0.80 |
| VLDL-c mg/dL | Total protein (g) | 0.10 | 0.62 |
| Triglycerides mg/dL | Total protein (g) | 0.10 | 0.61 |
| Non-HDL-c mg/dL | Total protein (g) | 0.10 | 0.63 |
| Total Cholesterol mg/dL | Total carbohydrate (g) | -0.08 | 0.67 |
| HDL-c mg/dL | Total carbohydrate (g) | 0.12 | 0.53 |
| LDL-c mg/dL | Total carbohydrate (g) | 0.16 | 0.43 |
| VLDL-c mg/dL | Total carbohydrate (g) | -0.16 | 0.43 |
| Triglycerides mg/dL | Total carbohydrate (g) | -0.12 | 0.56 |
| Non-HDL-c mg/dL | Total carbohydrate (g) | -0.10 | 0.61 |
| Total Cholesterol mg/dL | Total fiber (g) | -0.09 | 0.66 |
| HDL-c mg/dL | Total fiber (g) | 0.09 | 0.64 |
| LDL-c mg/dL | Total fiber (g) | -0.19 | 0.34 |
| VLDL-c mg/dL | Total fiber (g) | 0.14 | 0.48 |
| Triglycerides mg/dL | Total fiber (g) | 0.06 | 0.75 |
| Non-HDL-c mg/dL | Total fiber (g) | -0.10 | 0.61 |
| Total Cholesterol mg/dL | Monounsaturated fatty acids (g) | -0.05 | 0.81 |
| HDL-c mg/dL | Monounsaturated fatty acids (g) | 0.22 | 0.27 |
| LDL-c mg/dL | Monounsaturated fatty acids (g) | -0.12 | 0.55 |
| VLDL-c mg/dL | Monounsaturated fatty acids (g) | 0.14 | 0.48 |
| Triglycerides mg/dL | Monounsaturated fatty acids (g) | 0.01 | 0.96 |
| Non-HDL-c mg/dL | Monounsaturated fatty acids (g) | -0.11 | 0.58 |
| Total Cholesterol mg/dL | Saturated fatty acids (g) | -0.14 | 0.48 |
| HDL-c mg/dL | Saturated fatty acids (g) | 0.21 | 0.28 |
| LDL-c mg/dL | Saturated fatty acids (g) | -0.15 | 0.43 |
| VLDL-c mg/dL | Saturated fatty acids (g) | -0.04 | 0.84 |
| Triglycerides mg/dL | Saturated fatty acids (g) | -0.08 | 0.68 |
| Non-HDL-c mg/dL | Saturated fatty acids (g) | -0.20 | 0.31 |
| Total Cholesterol mg/dL | Trans fatty acids (g) | -0.21 | 0.29 |
| HDL-c mg/dL | Trans fatty acids (g) | **-0.38** | **0.04** |
| LDL-c mg/dL | Trans fatty acids (g) | -0.16 | 0.41 |
| VLDL-c mg/dL | Trans fatty acids (g) | -0.07 | 0.74 |
| Triglycerides mg/dL | Trans fatty acids (g) | 0.02 | 0.91 |
| Non-HDL-c mg/dL | Trans fatty acids (g) | -0.16 | 0.42 |
| Total Cholesterol mg/dL | Cholesterol (g) | -0.04 | 0.82 |
| HDL-c mg/dL | Cholesterol (g) | -0.18 | 0.37 |
| LDL-c mg/dL | Cholesterol (g) | 0.04 | 0.82 |
| VLDL-c mg/dL | Cholesterol (g) | 0.06 | 0.79 |
| Triglycerides mg/dL | Cholesterol (g) | 0.01 | 0.94 |
| Non-HDL-c mg/dL | Cholesterol (g) | -0.02 | 0.92 |
| Total Cholesterol mg/dL | Polyunsaturated fatty acids (g) | -0.15 | 0.46 |
| HDL-c mg/dL | Polyunsaturated fatty acids (g) | 0.05 | 0.81 |
| LDL-c mg/dL | Polyunsaturated fatty acids (g) | -0.17 | 0.40 |
| VLDL-c mg/dL | Polyunsaturated fatty acids (g) | 0.02 | 0.93 |
| Triglycerides mg/dL | Polyunsaturated fatty acids (g) | 0.02 | 0.91 |
| Non-HDL-c mg/dL | Polyunsaturated fatty acids (g) | -0.16 | 0.42 |
| Total Cholesterol mg/dL | Phytosterols (mg) | 0.02 | 0.92 |
| HDL-c mg/dL | Phytosterols (mg) | **0.59** | **<0.01** |
| LDL-c mg/dL | Phytosterols (mg) | -0.07 | 0.72 |
| VLDL-c mg/dL | Phytosterols (mg) | -0.08 | 0.71 |
| Triglycerides mg/dL | Phytosterols (mg) | -0.03 | 0.87 |
| Non-HDL-c mg/dL | Phytosterols (mg) | -0.07 | 0.72 |

F&V, Fruits/legumes/Vegetables; HDL-c, High-Density Lipoprotein; LDL-c, Low-Density Lipoprotein; VLDL-c, Very-Low-Density Lipoprotein; HFSS, High Fat/Sugar/Sodium. Bold numbers represent results with statistical significance (p < 0.05).

**Appendix C.1** Linear regression model for HDL-c changes (post/pre ratio) considering adjusted trans fatty acids intake to age, BMI, and lipid-lowering therapy before and after surgery.

|  | **Beta** | **95% CI** | | **Standard Error** | ***t*-value** | **p-value** |
| --- | --- | --- | --- | --- | --- | --- |
|  |  | **Inferior** | **Superior** |  |  |  |
| **Intercept** | 1.29 | 1.00 | 1.58 | 0.14 | 9.38 | <0.001 |
| **Trans-fat acid intake (g) (Post/Pre)** | -0.45 | -0.84 | -0.06 | 0.19 | -2.37 | 0.027 |
| **Age (10 years increase) - Pre** | -0.04 | -0.17 | 0.09 | 0.06 | -0.62 | 0.542 |
| **BMI (10 units increase) - Pre** | -0.04 | -0.18 | 0.10 | 0.07 | -0.63 | 0.533 |
| **LLT - before, but not after** | -0.14 | -0.36 | 0.08 | 0.11 | -1.29 | 0.211 |
| **LLT – before and after** | -0.09 | -0.34 | 0.15 | 0.12 | -0.79 | 0.435 |

Grouping as follows: not taking LLT before and after surgery (reference); taking LLT before, but not after surgery; taking LLT before and after surgery. LLT, Lipid-Lowering Therapy.

**Appendix C.2** Linear regression model for HDL-c changes (post/pre ratio) considering adjusted phytosterols intake to age, BMI, and lipid-lowering therapy before and after surgery

|  | **Beta** | **95% CI** | | **Standard Error** | ***t*-value** | **p-value** |
| --- | --- | --- | --- | --- | --- | --- |
|  |  | **Inferior** | **Superior** |  |  |  |
| **Intercept** | 0.72 | 0.50 | 0.94 | 0.10 | 6.85 | <0.001 |
| **Phytosterols intake (mg) (Post/Pre)** | 0.34 | 0.10 | 0.59 | 0.12 | 2.88 | 0.009 |
| **Age (10 years increase) ‒ Pre** | -0.03 | -0.16 | 0.10 | 0.06 | -0.46 | 0.648 |
| **BMI (10 units increase) ‒ Pre** | 0.03 | -0.11 | 0.18 | 0.07 | 0.45 | 0.654 |
| **LLT ‒ before, but not after** | 0.03 | -0.17 | 0.24 | 0.10 | 0.35 | 0.73 |
| **LLT – before and after** | 0.03 | -0.21 | 0.28 | 0.12 | 0.28 | 0.784 |

Grouping as follows: not taking LLT before and after surgery (reference); taking LLT before, but not after surgery; taking LLT before and after surgery. LLT, Lipid-Lowering Therapy.

**Appendix C.3** Linear regression model for HDL-c changes (post/pre ratio) considering adjusted trans fatty acids intake to age, BMI, and change in lipid-lowering therapy.

|  | **Beta** | **95% CI** | | **Standard Error** | ***t*-value** | **p-value** |
| --- | --- | --- | --- | --- | --- | --- |
|  |  | **Inferior** | **Superior** |  |  |  |
| **Intercept** | 1.26 | 0.99 | 1.52 | 0.13 | 9.81 | <0.001 |
| **Trans-fat intake (g) (Post/Pre)** | -0.42 | -0.80 | -0.04 | 0.18 | -2.29 | 0.031 |
| **Age (10 years increase) ‒ Pre** | -0.06 | -0.18 | 0.07 | 0.06 | -0.93 | 0.362 |
| **BMI (10 units increase) ‒ Pre** | -0.03 | -0.17 | 0.10 | 0.07 | -0.53 | 0.601 |
| **Change in LLT** | -0.10 | -0.29 | 0.08 | 0.09 | -1.14 | 0.267 |

BMI, Body Mass Index; LLT, Lipid-Lowering Therapy.

**Appendix C.4** Linear regression model for HDL-c changes (post/pre ratio) considering adjusted phytosterols intake to age, BMI, and change in lipid-lowering therapy.

|  | **Beta** | **95% CI** | | **Standard Error** | ***t*-value** | **p-value** |
| --- | --- | --- | --- | --- | --- | --- |
|  |  | **Inferior** | **Superior** |  |  |  |
| **Intercept** | 0.74 | 0.55 | 0.93 | 0.09 | 8.01 | <0.001 |
| **Phytosterols intake (mg) (Post/Pre)** | 0.33 | 0.10 | 0.56 | 0.11 | 2.94 | 0.007 |
| **Age (10 years increase) ‒ Pre** | -0.02 | -0.14 | 0.10 | 0.06 | -0.36 | 0.723 |
| **BMI (10 units increase) ‒ Pre** | 0.02 | -0.11 | 0.16 | 0.07 | 0.34 | 0.737 |
| **Change in LLT** | 0.01 | -0.17 | 0.18 | 0.08 | 0.06 | 0.951 |

BMI, Body Mass Index; LLT, Lipid-Lowering Therapy.

**Supplementary S1** Foods High in saturated Fats, Sodium, and added Sugars of the non-protective group (HFSS).

| **High in saturated fatty acids (6g/100g solids; 3g/100mL liquids)** | **High in added sugars (15g/100g solids; 7.5g/100mL liquids)** | **High in sodium (600mg/100g solids; 300mg/100mL liquids)** |
| --- | --- | --- |
| Margarine | Sweetened ready-to-drink teas | Salted margarine |
| Cheeses (parmesan, roquefort, provolone, gorgonzola, coalho) | Sweetened condensed milk | Japanese peanuts |
| Cream cheese and "requeijão" | Sweet biscuits (cornstarch, coconut, chocolate) | Black and green olives |
| Processed meats (sausages, jerked beef, mortadella, hot dog, bacon, salami, pork fat) | Candied fruits | Codfish |
| Chocolate and candy bars | Soft drinks | Processed meats (sausages, jerked beef, mortadella, sausage, bacon, salami, pork fat, turkey breast, ham) |
| packaged cakes | Sweetened ready-to-drink juices | Traditional mayonnaise |
| Hamburger | Packaged cakes and pies | Bottled sauces (mustard, Worcestershire, tomato) |
| Stock cube | Chocolate and candy bars | Cheese bread (pão de queijo) |
| Heavy cream | Dulce de leche | Bread (pão francês) |
| Sweet filled cookies | Flavored gelatins | Cheeses (parmesan, roquefort, provolone, gorgonzola, coalho) |
| Ice cream | Sweetened breakfast cereals | Cream cheese and "requeijão" |
| Potatoes chips | Candies (mint, banana, toffee, gummy, hard candy) | Packaged corn snacks |
| Coconut milk | Stock cube | Canned sardines |
| Cheese bread (pão de queijo) | Chocolate milk powder | Packaged meat 'sfiha' |
|  | Ice cream | Salty crackers |
|  | Ketchup | Stock cube |
|  | Fruit jam | Instant noodles with seasoning |
|  | Infant cereal with milk | Packaged lasagna with bolognese sauce |
|  | Petit Suisse cheese | Potatoes chips |
|  | Banana sweet bar |  |
|  | Peanut candy (paçoca) |  |
|  | Protein bars |  |

The non-protective group (HFSS) comprised foods high in saturated fats, sodium, and added sugars, as defined the Brazilian nutritional labeling legislation.
